# High Diversity and Spatiotemporal Dynamics of Silica-Scaled Chrysophytes (Class Chrysophyceae) in Reservoirs of the Angara Cascade of Hydroelectric Dams

**DOI:** 10.3390/biology14101325

**Published:** 2025-09-25

**Authors:** Anna Bessudova, Yuri Galachyants, Alena Firsova, Artyom Marchenkov, Andrey Tanichev, Darya Petrova, Yelena Likhoshway

**Affiliations:** Limnological Institute, Siberian Branch of the Russian Academy of Sciences, 3 Ulan-Batorskaya, 664033 Irkutsk, Russialikhoshway@mail.ru (Y.L.)

**Keywords:** freshwater, river regulation, dams, reservoirs, diversity, silica-scaled chrysophytes, sustainability

## Abstract

The study of aquatic biodiversity in the context of ecosystem sustainability is of urgent research importance, with several existing knowledge gaps. Among the under-studied groups are silica-scaled chrysophytes. Their cells are covered with silica scales and bristles/spines, the species-specific structure of which can be distinguished only by electron microscopy. In June and August 2024, samples were collected from a broad aquatic system comprising the southern part of Lake Baikal and a cascade of four reservoirs formed after the construction of hydroelectric dams on the Angara River flowing from Lake Baikal. Using electron microscopy, we identified 45 species of silica-scaled chrysophytes in phytoplankton in 2024, and the overall checklist was expanded to 57, accounting for interannual differences. Clear differences in species composition and richness were observed both between seasons and among reservoirs. Approximately a quarter of the recorded species were heterotrophs, which do not contribute to primary production, whereas 44% were phototrophs and 31% mixotrophs, both groups contributing to the Si cycle and to primary production. Continuous monitoring of reservoirs is essential for understanding the processes shaping silica-scaled chrysophytes diversity and may serve as an additional criterion for assessing the sustainability and transformation of freshwater ecosystems.

## Simple Summary

One of the human impacts on nature is the construction of hydroelectric dams. As a result, the river expands their channels, the flow slows down, artificial reservoirs are formed, and new or altered habitat conditions for aquatic organisms are created. Using electron microscopy, we examined phytoplankton samples from a vast aquatic system—including the southern part of Lake Baikal, the Angara River flowing from it, and a cascade of four reservoirs—and found a high species richness of silica-scaled chrysophytes. The species composition of these microscopic organisms varied both among reservoirs and between seasons. Our results are important for a better understanding of the processes driving diversity formation and the transformation of aquatic ecosystems under human influence.

## 1. Introduction

The central task of biology is to document the biodiversity of organisms, to understand the mechanisms of its formation, and develop strategy for its preservation. Among microeukaryotes with flagella, silica-scaled chrysophytes represent a distinctive group that is widespread in freshwater ecosystems [1,2,3].

Several studies have shown that silica-scaled chrysophytes may constitute a substantial component of the plankton and surface sediments in modern reservoirs [4,5]. Consequently, they play an important role both in the Si cycle and in primary production. Because many species occur along distinct environmental gradients, they serve as valuable bioindicators in ecological and paleolimnological research [6,7,8,9,10,11,12]. Temperature, pH, and electrical conductivity are considered the most influential environmental drivers of their distribution [11]. Silica-scaled chrysophytes also differ in trophic strategies: members of the genera *Mallomonas* Perty and *Synura* Ehrenberg are autotrophs [13,14]; *Chrysosphaerella* Lauterborn and *Spiniferomonas* Takahashi are regarded as mixotrophs [14,15], while *Paraphysomonas* De Saedeleer and *Lepidochromonas* Kristiansen are heterotrophs [13,14,16]. These ecological differences further underscore the importance of studying their diversity for understanding freshwater ecosystem functioning.

Knowledge of the distribution of silica-scaled chrysophytes remains incomplete, largely due to difficulties in reliable species identification. These organisms can sometimes be recognized in living phytoplankton samples, but this is not always feasible. More often, identification relies on the species-specific morphology of siliceous elements that persist after cell lysis in fixed sample. Such structural features can only be observed with scanning (SEM) or transmission (TEM) electron microscopy, techniques that are not universally accessible to all researchers.

Currently, the species concept of silica-scaled chrysophytes is based on the ultrastructural features of silica elements that densely cover the cell surface—scales ranging in size from 0.2 nm to 8 μm and bristles/spines—which is consistent with molecular data [11,16,17]. Thus, SEM and TEM remain indispensable tools for assessing the diversity of silica-scaled chrysophytes.

River impoundment for hydroelectric power generation profoundly alters natural aquatic ecosystems, often reshaping the diversity of resident organisms. On the Angara River, which flows from Lake Baikal, four large reservoirs have been created: Irkutsk, Bratsk, Ust-Ilimsk, and Boguchany. In this study, we examined the diversity of silica-scaled chrysophytes in these artificial lakes. We hypothesized that, because the river extends more than 1335 km from its source to the downstream Boguchany Reservoir, with reservoirs differing in depth and shoreline complexity, the species composition and richness of silica-scaled chrysophytes would be heterogeneous across environmental gradients in this large regulated river system. To test this hypothesis, we sampled during two seasons in 2024—the hydrological spring (June) and the summer (August)—identified species of silica-scaled chrysophytes using SEM and TEM, and analyzed their distribution across reservoir ecosystems.

## 2. Materials and Methods

### 2.1. Site Description

The study area covers Southern Baikal and four reservoirs on the Angara River, created through impoundment for hydroelectric power generation: Irkutsk, Bratsk, Ust-Ilimsk and Boguchany. The climate in the study area is sharply continental, and the landscape is dominated by taiga forest.

Lake Baikal is the world’s deepest (maximum depth of 1642 m) freshwater and oligotrophic lake, containing about 20% of the planet’s liquid freshwater reserves [18]. Its surface area is 31,500 km^2^, with a mean depth of 758 m. Although the lake is cold-water, the surface water layer in the pelagic zone in southern part of Lake Baikal can warm up to 16.2 °C in summer [19]. The waters of Baikal belong to the bicarbonate class of the calcium group, and are characterized by very low mineralization (≤100 mg∙m^−3^), low organic matter content, and high concentrations of dissolved oxygen [18]. The Angara River flows out of the lake’s southern basin.

Throughout its course, the Angara remains strongly influenced by Baikal water; even near its confluence with the Yenisei River, Baikal still contributes about 45% of the total discharge [20]. The river length from its source to the Yenisei is 1779 km.

Irkutsk Reservoir. The first reservoir in the Angara Cascade extends for 56 km from the outflow of Lake Baikal to the dam located within the city of Irkutsk. It was filled to normal pool between 1956 and 1962 [21]. The reservoir lies at 456–457 m above sea level, covers 154 km^2^, has a maximum depth of 35 m, and the average depth of 13.6 m. The ice period lasts an average of 145 days, with the maximum ice thickness up to 100 cm [21,22]. At the Baikal outlet and immediately downstream of the dam, the river never freezes; winter water temperature ranges from 0.3 to 1.7 °C. The reservoir’s climate is strongly influenced by proximity to Lake Baikal, making it milder than downstream areas. Its thermal regime is heterogeneous: during the period of open water, the dammed section warms up to 17.3 °C and bays up to 21 °C [19,23]. The hydrochemical regime largely reflects the composition of Baikal water [24].

Bratsk Reservoir. Filled between 1961 and 1967, the Bratsk Reservoir lies at 392–403 m above sea level, covers 5470 km^2^, reaches 150 m in maximum depth, and has the average depth 31.1 m [21,22]. The ice period averages 180 days, with maximum ice thickness up to 135 cm. More than a half of the inflow (62–65%) derives from Baikal water. Hydrologically and hydrobiologically, the reservoir is divided into three main sections named after the river valleys that form them: Iya, Oka, and Angara [25]. During the open water period, surface temperatures in Bratsk Reservoir do not exceed 20 °C.

Ust-Ilimsk Reservoir. This reservoir was filled between 1974 and 1977 to its normal pool. It lies at 294.5–296 m above sea level, has a surface area of 1922 km^2^, a maximum depth of 97 m, and an average depth of 30.7 m [22,26]. The ice period averages 183 days.

Boguchany Reservoir. Filled between 2012 and 2015, accounting for pauses, the Boguchany Reservoir lies at 208 m above sea level, covers 2326 km^2^, has a maximum depth of 70 m, and an average depth of 25 m [27]. The ice period lasts about 183 days, with the maximum ice thickness up to 115 cm.

### 2.2. Sampling and Microscopy

**Figure 1:**
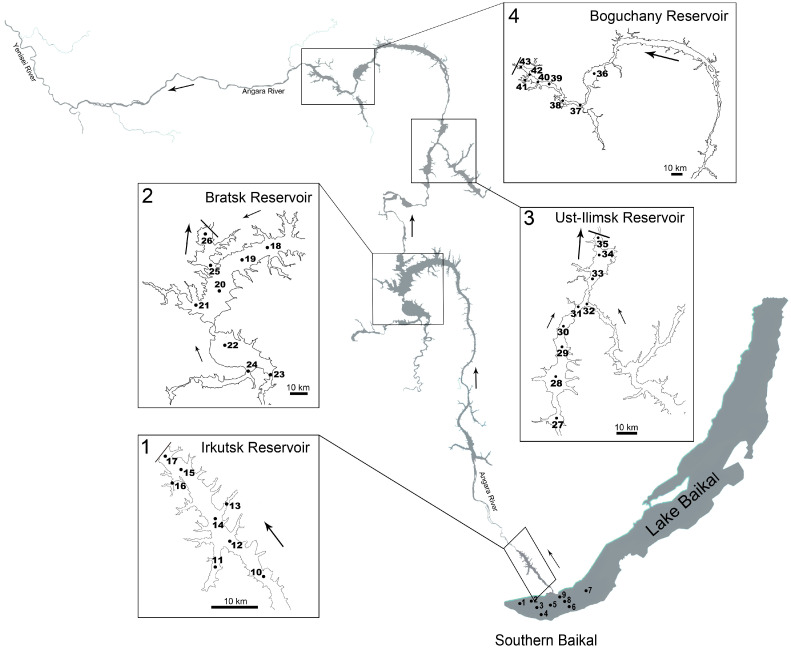
Sampling map of Southern Baikal and Angara Cascade reservoirs. Numbers indicate sampling stations; arrows show the direction of water flow.

Plankton samples were collected during filed expeditions of the RSF project “Communities of microeukaryotes in Angara Cascade reservoirs” in two seasons of 2024: hydrological spring and summer. In Southern Baikal (SB), samples were collected on 24–25 May and 11 August; in the reservoirs of the Angara Cascade—Irkutsk (IR), Bratsk (BrR), Ust-Ilimsk (UR) and Boguchany (BgR)—sampling was conducted on 3–11 June and 23–31 August (Figure 1; Table S1).

Integral samples (1.2 L) were obtained by pooling equal volumes of water collected from depths of 0, 5, and 10 m. These were filtered through polyethylene terephthalate track-etched membranes (Reatrek, Obninsk, Russia) with a pore size of 2 μm using a PVF-47/3 filtration unit (Vladisart, Vladimir, Russia). After filtration, each membrane was placed into a 50 mL Falcon tube with 45 mL of unfiltered water; the retained material was rinsed from the filter with 5 mL of the same water and fixed with a formaldehyde (final concentration of 3.7–4%).

For analysis of species richness of silica-scaled chrysophytes, samples were washed to remove the fixative. One milliliter of sample was transferred into a 1.5 mL Eppendorf tube and centrifuged in a MiniSpin centrifuge (Eppendorf, Humburg, Germany) at 13,400 rpm for 10 min. The supernatant was removed, and the pellet was resuspended in distilled water and centrifuged three times. The washed pellet was treated with 30% hydrogen peroxide (H_2_O_2_) and incubated at 80 °C for 2–4 h, then rinsed free of H_2_O_2_ with distilled water, through three additional centrifugations. The final suspension was used for SEM or TEM.

For SEM, 50 µL of washed suspension was mixed thoroughly by shaking, and a droplet was on an SEM stub pre-cleaned with ethanol. The stub ID was recorded in the laboratory log. Samples were air-dried, sputter-coated with gold using a vacuum sputtering unit SCD 004 (Balzers, Liechtenstein), and examined using Quanta 200 SEM (FEI Company, Hillsboro, OR, USA). For TEM, material was deposited on a 3 mm circle of mesh coated with a formvar film and allowed to dry at room temperature. Observations were performed with LEO 906E TEM (Carl Zeiss, Jena, Germany).

To estimate the relative abundance of silica-scaled chrysophytes, 20 mL of unfixed sample was filtered onto track-etched membrane (Reatrek, Obninsk, Russia) with a pore size of 3 µm using a syringe with custom nozzle. Each filter was then rinsed with 20 mL of 70% ethanol mounted on SEM stubs with double-sided tape, air-dried, and examined with Quanta 200 SEM. This categorical assessment of abundance was not used in formal statistical analyses but served only as an approximate measure to identify the most frequently occurring silica-scaled chrysophytes taxa.

### 2.3. Geographical Distribution

We analyzed the geographical distribution of silica-scaled chrysophytes in the studied reservoirs and assigned taxa to the previously proposed latitudinal and longitudinal groups of geographical distribution [1,2,3], according to their preferred climatic zones and geographical ranges. The latitudinal groups comprised P (polyzonal)—species occurring across all climatic zones; A-Bor (arctic–boreal)—species restricted to the temperate and/or Arctic zones of the Northern Hemisphere; and B (boreal)—species confined to the boreal zone. The longitudinal groups comprised End (endemic)—species with a geographically limited continental range; K (cosmopolitan)—species recorded on all six continents; W (widespread)—species occurring on one or two continents; R (scattered/rare)—species rarely found and patchily distributed across different latitudes. The “UK” group includes species with unknown geographical distribution, those identified only to the genus level or taxa showing atypical morphology.

### 2.4. Statistical Analysis

We analyzed Secchi depth (S, m), pH, temperature (T, °C), and electrical conductivity (EC, mS∙m^−1^) with two-factor ANOVA implemented as linear models of the form “response ~ Season * Region”, where Season had two levels (spring, summer) and Region had five levels (SB, IR, BrR, UR, BgR). Because sample sizes across Season × Region cells were slightly unbalanced, we computed Type-II sums of squares using the car::Anova procedure in R. Model assumptions were checked by visual inspection of residuals (Q–Q and scale–location plots) and by formal tests (Shapiro–Wilk on model residuals, Levene’s test for homogeneity of variance, car::leveneTest). No severe violations were detected, so responses were analyzed on the original scale without transformation.

When the Season × Region interaction was significant, we interpreted simple effects using estimated marginal means (emmeans package). Specifically, we compared (i) Seasons within each Region and (ii) Regions within each Season. Pairwise contrasts were adjusted for multiple comparisons with Tukey’s method (Tukey–Kramer where group sizes differed), and we report contrast estimates (Δ) with standard errors, *t*-ratios, and Tukey-adjusted *p*-values. All analyses were conducted in R (packages car v.3.1.3, emmeans v.1.11.2, broom v.1.0.9 ggplot2 v.3.5.2).

Community composition was analyzed from presence/absence data. This reflects the nature of the dataset: electron microscopy (SEM/TEM) provides reliable information on species occurrence, but not quantitative abundances, since fragile scaled cells are often destroyed during sample preparation, leaving only fragments. Robust abundance data are therefore unattainable, making presence/absence the most consistent basis for comparing distributions of silica-scaled chrysophytes across sites and seasons.

Pairwise community dissimilarities between samples were calculated using the Bray–Curtis index, which is widely applied in ecological studies to quantify compositional differences. The resulting dissimilarity matrix was then used for complementary exploratory analyses.

Principal Coordinates Analysis (PCoA) was applied to visualize the major gradients in community composition. PCoA projects multivariate dissimilarities into a reduced space, facilitating graphical representation of seasonal and regional assemblages. To evaluate relationships with environmental gradients, we fitted abiotic variables (temperature, pH, electrical conductivity, water transparency) onto the ordination using the envfit procedure in the vegan package v.2.5-6 [28], with significance assessed by 999 permutations. This identifies the factors most strongly associated with variation in community structure.

Finally, communities were clustered using affinity propagation (R package apcluster v.1.4.13 [29]). Unlike classical hierarchical methods, affinity propagation does not require predefining the number of clusters; instead, it identifies exemplar samples and builds clusters around them, providing an objective basis for distinguishing seasonal and regional assemblages.

Together, these steps—dissimilarity calculation, ordination, environmental fitting, and clustering—constitute an exploratory framework for assessing the ecological structure of silica-scaled chrysophytes communities in Lake Baikal and the Angara Cascade reservoirs.

## 3. Results

### 3.1. Water Parameters

**Figure 2:**
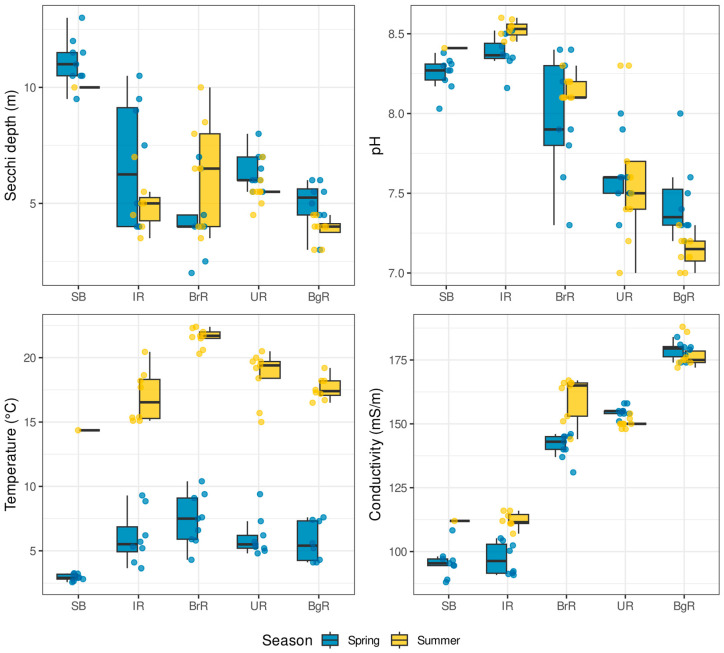
Abiotic factors by region and season. Boxplots show Secchi depth, pH, temperature, and electrical conductivity across five regions of the Baikal–Angara Cascade (SB, IR, BrR, UR, BgR). The *x*-axis shows region, the *y*-axis variable values; colors denote season (spring vs. summer). Boxes represent medians and interquartile ranges, whiskers extend to 1.5 × IQR, and semi-transparent points are individual samples. Group differences were tested with two-way ANOVA (response ~ Season ∙ Region) followed by Tukey (Tukey–Kramer) post hoc tests; detailed contrasts and adjusted *p*-values are given in Table S1.

Across the cascade, temperature exhibited the strongest and most consistent seasonal signal (Figure 2; Table S1): in every region summer was warmer than spring by ~11–14 °C (all Tukey-adjusted *p* < 0.0001). In spring, SB was colder than all reservoirs by ~3–4.5 °C (e.g., SB–IR Δ = −3.10 °C, *p* = 0.0010; SB–BrR Δ = −4.46 °C, *p* < 0.0001; SB–UR Δ = −3.10 °C, *p* = 0.0006; SB–BgR Δ = −2.76 °C, *p* = 0.0043), while IR, BrR, UR, and BgR did not differ. In summer, SB remained coldest and BrR warmest (IR–BrR Δ = −4.58 °C, *p* < 0.0001; BrR–UR Δ = +2.94 °C, *p* = 0.0013; BrR–BgR Δ = +3.97 °C, *p* < 0.0001). Electrical conductivity also showed a clear pattern: at the upstream end (SB–BrR) it increased from spring to summer (Spring–Summer contrasts negative: SB Δ = −16.26 mS/m, *p* = 0.0037; IR Δ = −14.95 mS/m, *p* < 0.0001; BrR Δ = −18.89 mS/m, *p* < 0.0001), whereas UR and BgR showed no seasonal shift (*p* ≥ 0.058). Spatially, EC rose consistently downstream in both seasons (BgR > UR > BrR > IR > SB), with nearly all pairwise differences highly significant in spring and summer (*p* ≤ 0.0009), except for IR≈SB in summer. Together, these results indicate that temperature and mineralization represent the primary seasonal axes of environmental variation.

Water transparency (Secchi depth) varied both seasonally and regionally, but patterns differed among reservoirs. In IR, transparency was higher in spring than in summer (Δ = +1.76 m; *p* = 0.0256), whereas in BrR it was higher in summer than in spring (Δ = +2.11 m; *p* = 0.0037); SB, UR, and BgR showed no seasonal difference. Spatial contrasts were pronounced: in spring SB was markedly clearer than all reservoirs by ~4.7–6.8 m (*p* < 0.03 versus each), and in summer SB remained the clearest, exceeding IR/UR/BgR by ~4.4–6.1 m (*p* ≤ 0.046). Among reservoirs in summer, BrR was clearer than BgR (Δ = +2.51 m; *p* = 0.0078). pH exhibited only minor seasonal changes limited to BgR where summer values were slightly lower than in spring (Δ = +0.31, *p* = 0.011). A consistent longitudinal gradient was observed: in spring, SB≈IR ≥ BrR > UR≈BgR (key contrasts *p* ≤ 0.015), and in summer SB≈IR > BrR > UR > BgR, with SB and IR indistinguishable.

### 3.2. Seasonal Dynamics of Silica-Scaled Chrysophytes

**Figure 3:**
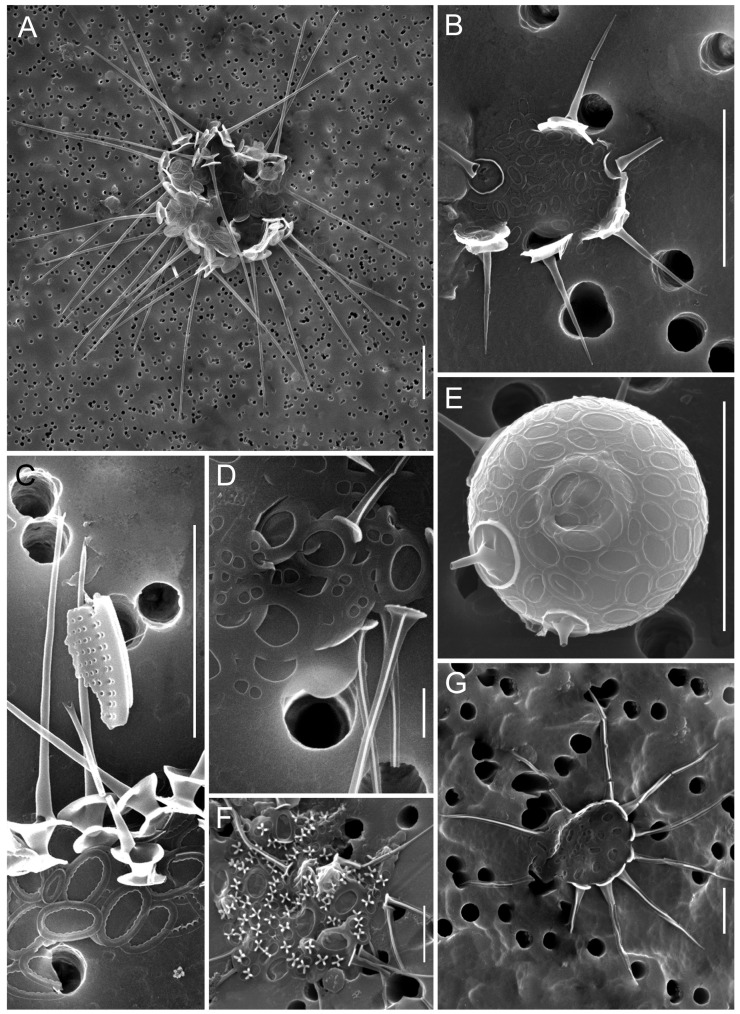
Micrographs of silica-scaled chrysophytes of the genera *Chrysosphaerella* and *Spiniferomonas*. *Chrysosphaerella baikalensis*, destroyed cells (**A**), *C. coronacircumspina*, destroyed cell (**B**), *C. coronacircumspina*, Stomatocyst 156, Zeeb & Smol 1993, covered with individual plate and spine scales (**E**), *C. brevispina*, individual plate scales and spines (**C**), *Spiniferomonas serrata*, individual plate and spine scales (**D**), *S. crucigera*, destroyed cell (**F**), *S. bilacunosa*, destroyed cell (**G**). SEM. Scale bars: (**D**,**F**,**G**)—2 µm; (**A**–**C**,**E**)—10 µm.

**Figure 4:**
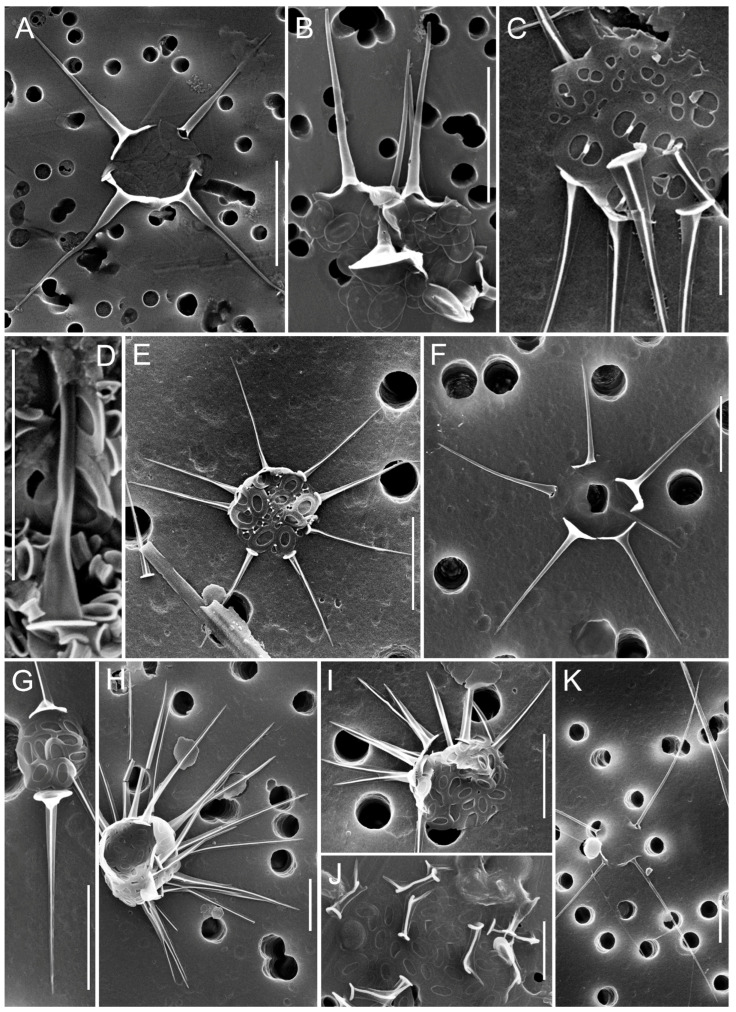
Micrographs of silica-scaled chrysophytes of the genus *Spiniferomonas*. *Spiniferomonas conica*, destroyed cells (**A**), *S. septispina*, individual plate and spine scales (**B**), *S. triangularis*, individual plate and spine scales (**C,D**), *S. cornuta*, destroyed cell (**E**), *S. bourrellyi*, destroyed cells (**F**,**K**), *S. silverensis*, destroyed cell (**G**), *S. cuspidata*, destroyed cell (**H**), *S. trioralis*, destroyed cell (**I**), *S. takahashii*, destroyed cell (**J**). SEM. Scale bars: (**C**,**J**)—2 µm; (**A**,**B**,**D**–**I**,**K**)—5 µm.

**Figure 5:**
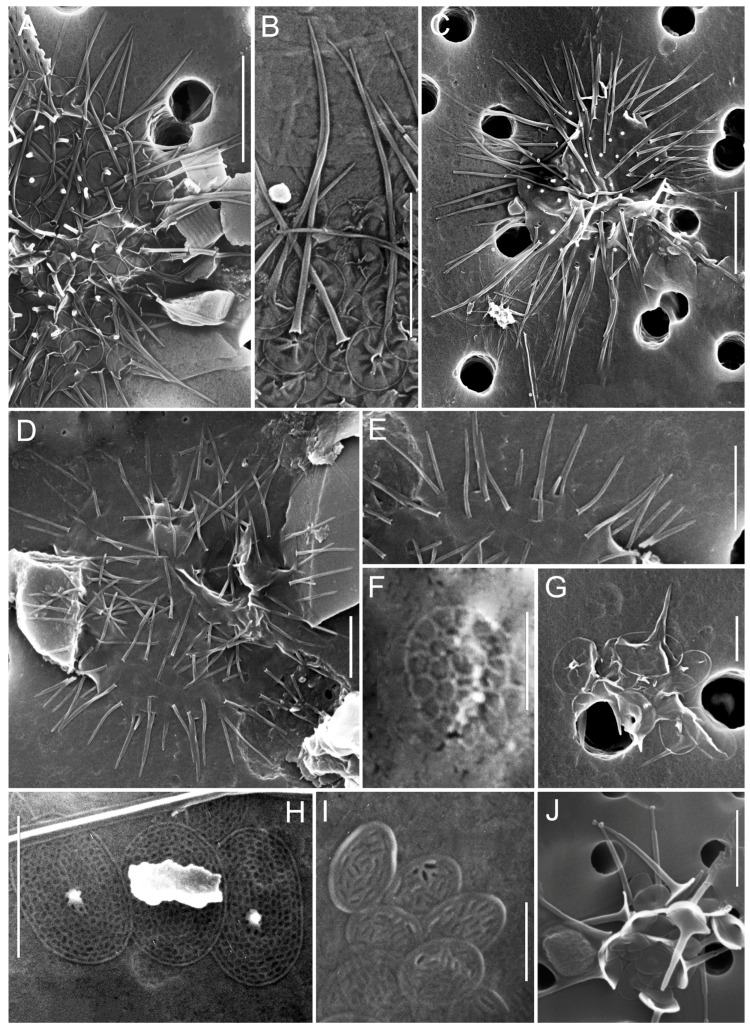
Micrographs of silica-scaled chrysophytes of the genera *Lepidochromonas* and *Paraphysomonas*. *Paraphysomonas* sp. 1, destroyed cell (**A**), *P. uniformis* subsp. *hemiradia*, individual nail-like spine scales (**B**), *P.* cf. *acuminata*, destroyed cell (**C**), *Paraphysomonas* sp. 3, destroyed cell (**D**,**E**), *P. butcheri*, individual coarsely perforated silica plate scales with additional structures in the form of a basket (**F**), *P. gladiata*, individual plate and spine scales (**G**), *Lepidochromonas takahashii*, individual plate scales (**H**), *P. vacuolata*, individual plate scales (**I**), *Paraphysomonas* sp. 4, individual plate and spine scales (**J**). SEM. Scale bars: (**F**,**I)**—1 µm; (**G**,**H**,**J**)—2 µm; (**A**–**I**)—5 µm.

**Figure 6:**
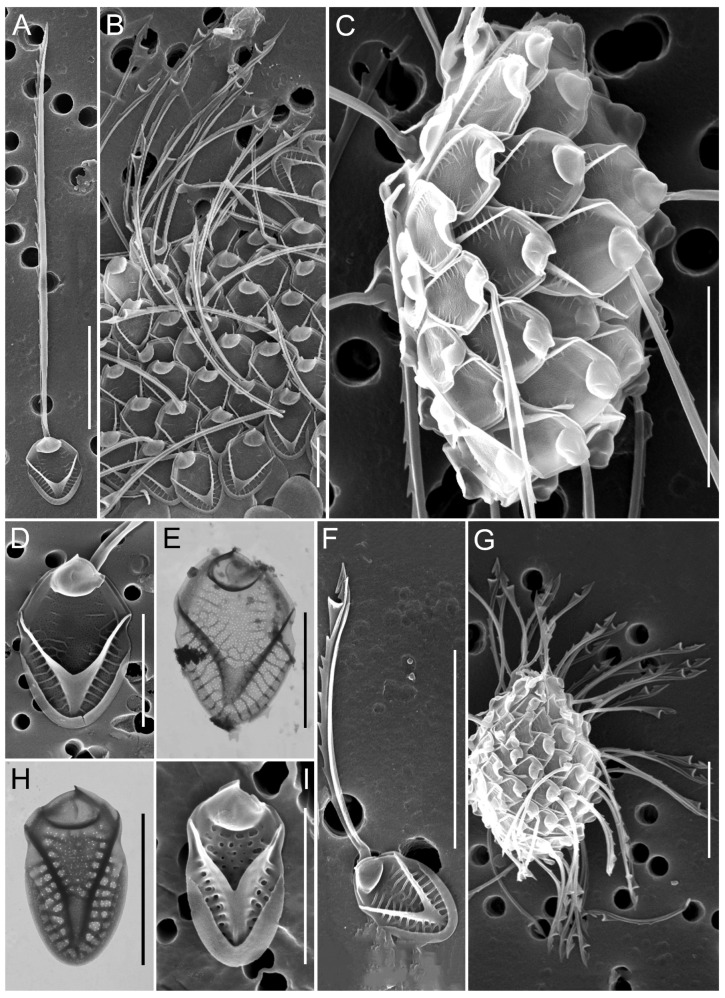
Micrographs of silica-scaled chrysophytes *Mallomonas acaroides* and *Mallomonas acaroides* forma. *Mallomonas acaroides*, scale with long serrated bristle (**A**), *M. acaroides*, scales with curved, helmet-shaped bristles (**B**), *M. acaroides*, whole cell covered with scales (**C**), *M. acaroides*, individual scales (**D**,**E**), *M. acaroides* forma, scale with curved, helmet-shaped bristles (**F**), *M. acaroides* forma, cell covered with scales and bristles (**G**), *M. acaroides* forma, individual scales (**H**,**I**). SEM (**A**–**D**,**F**,**G**,**I**), TEM (**E**,**H**). Scale bars: (**B**,**D**,**E**,**H**,**I**)—5 µm; (**A**,**C**,**F**,**G**)—10 µm.

**Figure 7:**
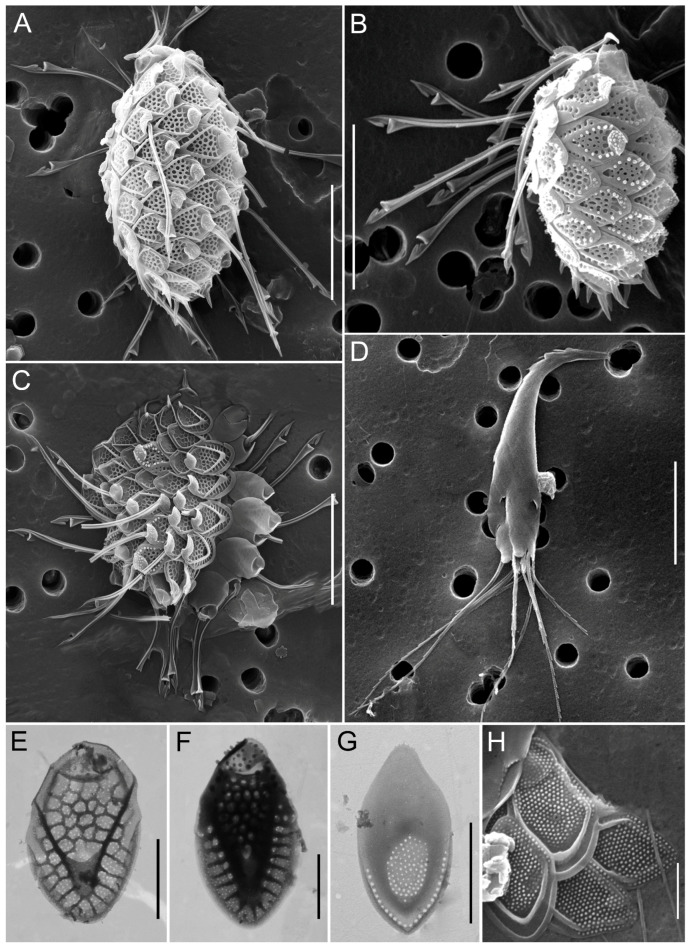
Micrographs of silica-scaled chrysophytes of the genus *Mallomonas*. *Mallomonas crassisquama*, cells covered with scales with papillae on the anterior submarginal ribs and on the V-rib and scales of typical morphology var. *crassisquama* (**A**–**C**), *Mallomonas crassisquama*, scales with papillae on the anterior submarginal ribs and scales of typical morphology var. *crassisquama* (**E**,**F**), *M. akrokomos*, cells covered with scales (**D**), *M. akrokomos*, individual scale (**G**), *M.* cf. *annulata*, individual scales (**H**). Scale bars: (**E**–**H**)—2 µm; (**A**–**D**)—10 µm.

**Figure 8:**
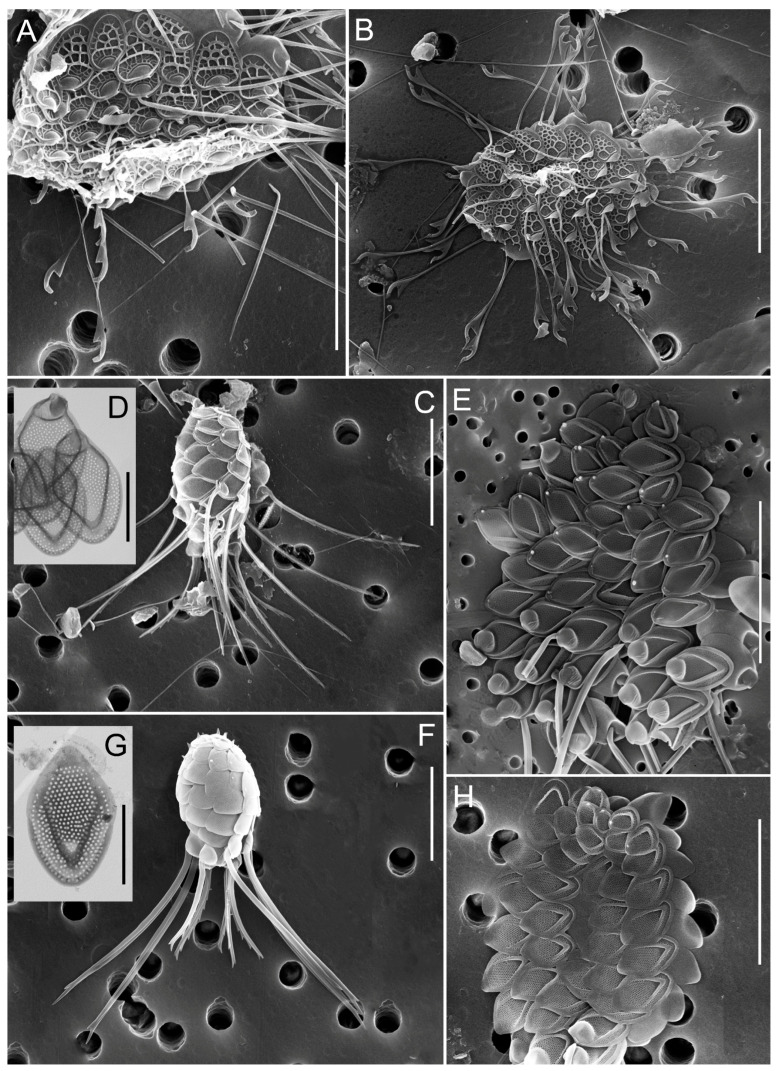
Micrographs of silica-scaled chrysophytes of the genus *Mallomonas*. *Mallomonas heterospina*, destroyed cell (**A**), *M. multiunca*, destroyed cell (**B**), *M. alpina*, cell covered with scales (**C**), *M. alpina*, individual scales (**D**,**E**), *M. tonsurata*, cell covered with scales (**F**), *M. tonsurata*, individual scales (**G**,**H**). Scale bars: (**D**,**G**)—2 µm; (**A**–**C**,**E**,**F**,**H)**—10 µm.

**Figure 9:**
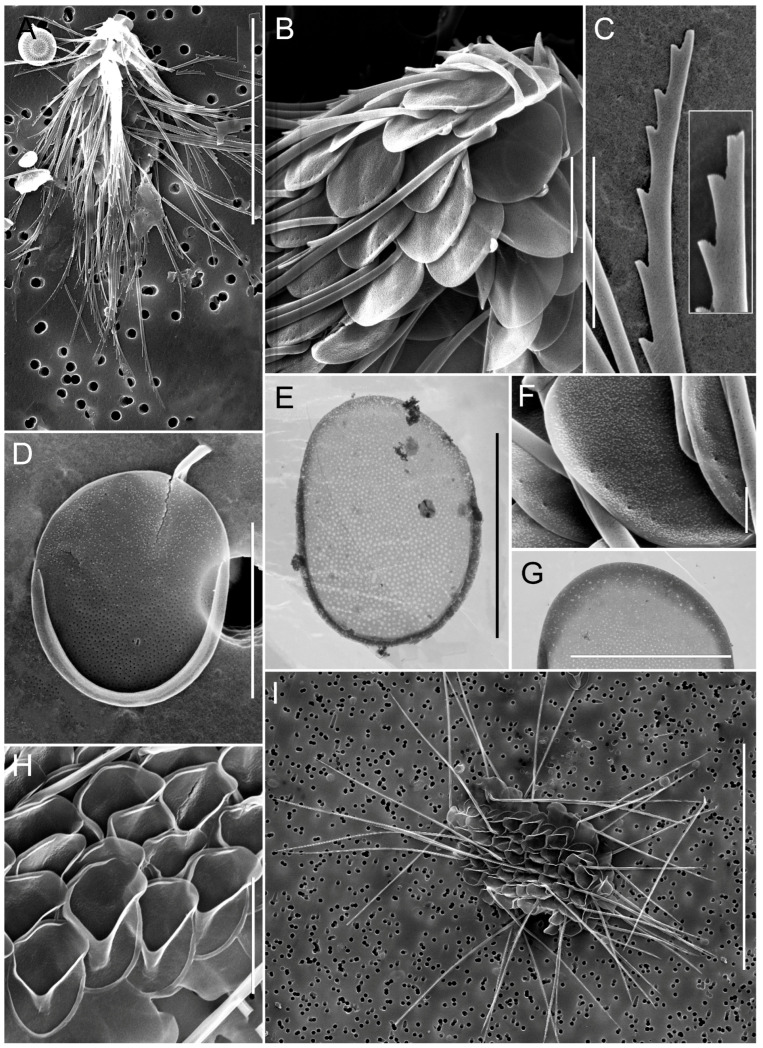
Micrographs of silica-scaled chrysophytes of the genus *Mallomonas*. *Mallomonas* cf. *caudata*, cell covered with scales (**A**), *M.* cf. *caudata*, the apical part of the cell is covered with scales with bristles extending from them (**B**), *M.* cf. *caudata*, the apex of the bristle (**C**), *M.* cf. *caudata*, individual scales (**D**,**E**), *M.* cf. *caudata*, large pores in the apical part of the scale (**F**), *M.* cf. *caudata*, apical part of the scale (**G**), *M. vannigera*, scales (**H**), *M. vannigera*, destroyed cell (**I**). Scale bars: (**F**)—1 µm; (**B**–**E**,**G**,**H**)—5 µm; (**I**)—40 µm; (**A**)—50 µm.

**Figure 10:**
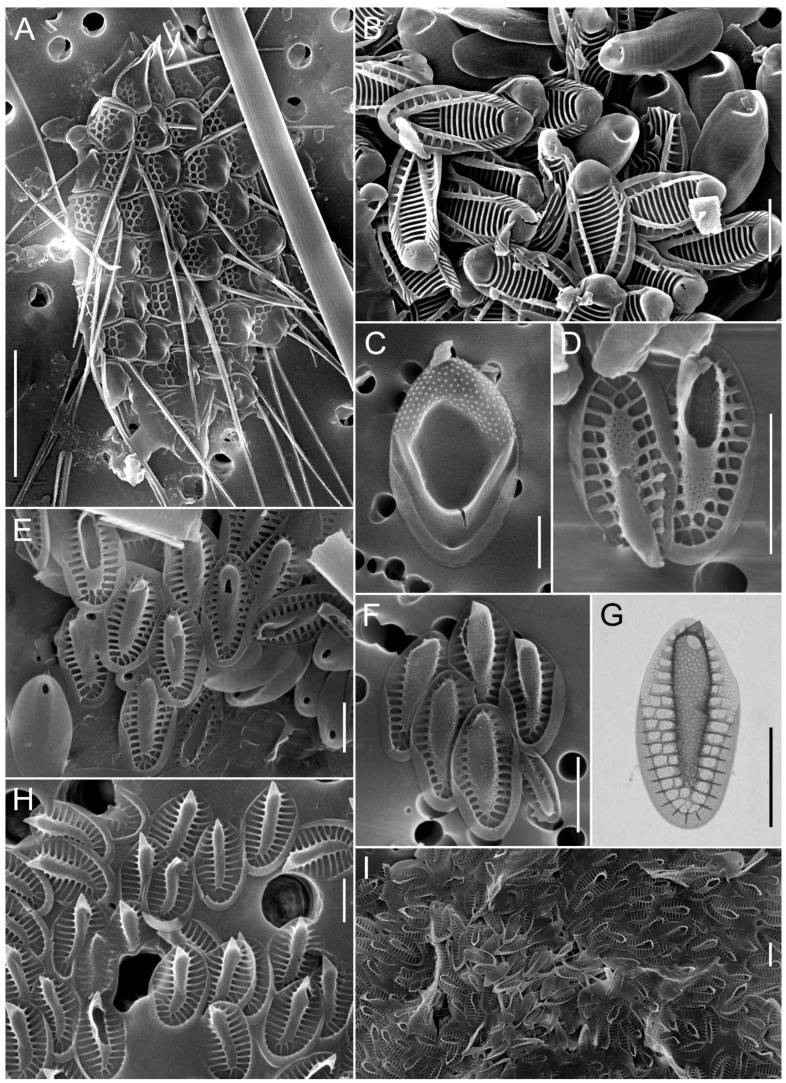
Micrographs of silica-scaled chrysophytes of the genera *Mallomonas* and *Synura*. *Mallomonas punctifera*, cell covered with scales (**A**), *M. striata*, scales (**B**), *M. insignis*, individual scale (**C**), *S. petersenii*, individual scales (**D**–**G**), *Synura glabra*, individual scales (**H**,**I**). Scale bars: (**B**–**I**)—2 µm; (**A**)—10 µm.

**Figure 11:**
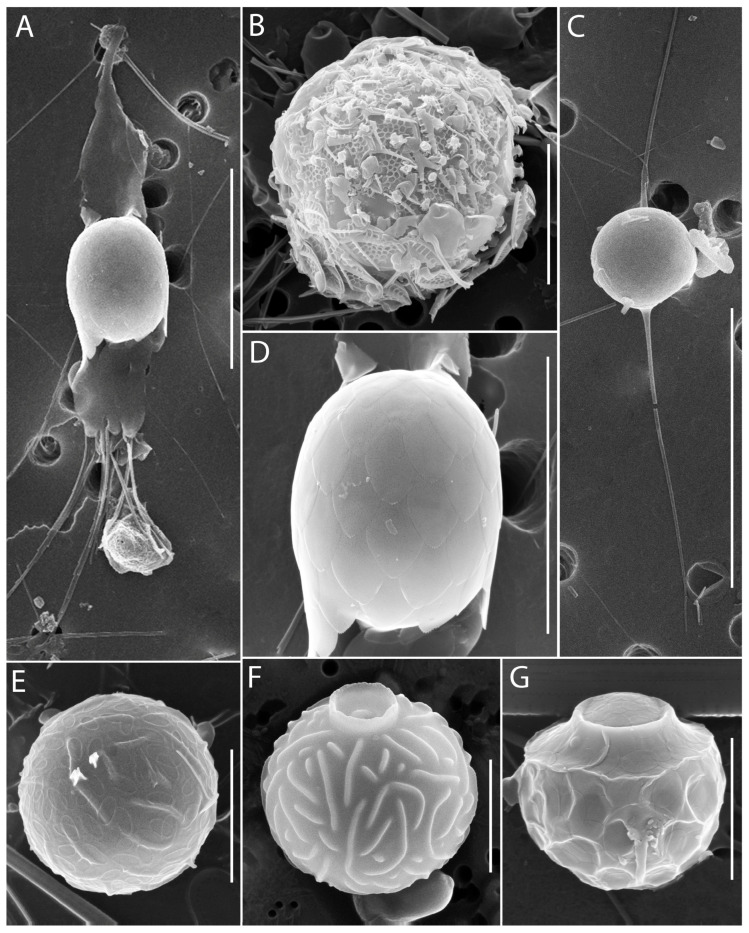
Micrographs of stomatocysts of silica-scaled chrysophytes. *Mallomonas akrokomos*, cell that formed the Stomatocyst 118, Zeeb et al. 1990 (**A**,**D**), *M. crassisquama*, Stomatocyst 166, Zeeb & Smol 1993, covered with scales (**B**), *S. bourrellyi*, cell that formed the stomatocyst (**C**), *S. trioralis*, Stomatocyst 111 Zeeb et al. 1990, covered with plate scales (**E**,**F**), *S. bourrellyi*, Stomatocyst 180, Zeeb & Smol 1993, covered with plate scales (**G**). Scale bars: (**E**–**G**)—5 µm; (**B**,**D**)—10 µm; (**A**)—15 µm; (**C**)—20 µm.

In 2024, a total of 45 species of silica-scaled chrysophytes were identified in SB and the reservoirs of the Angara Cascade (Figure 3, Figure 4, Figure 5, Figure 6, Figure 7, Figure 8, Figure 9, Figure 10 and Figure 11; Table S2). Species richness varied markedly among regions: IR harbored the highest diversity (33 species), followed by BrR and BgR (25 species each), while SB and UR were poorest, with 11 and 10 species, respectively.

**Figure 12:**
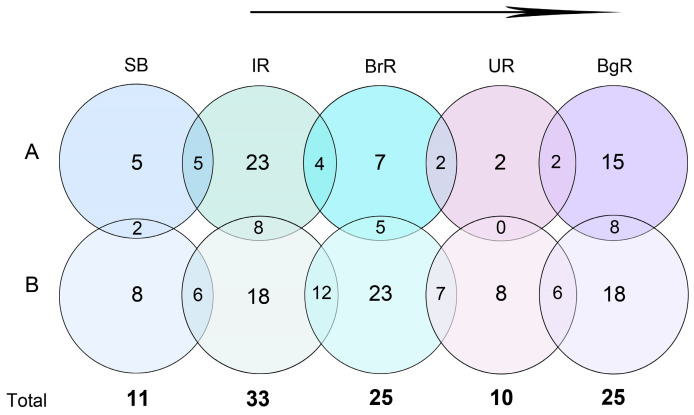
A Venn diagram showing the relationship of silica-scaled chrysophytes richness between SB and Angara Cascade reservoirs by seasons (A—spring; B—summer). The total number of species in each reservoir in 2024 is also shown below. The arrow indicates the flow direction of the Angara River.

**Figure 13:**
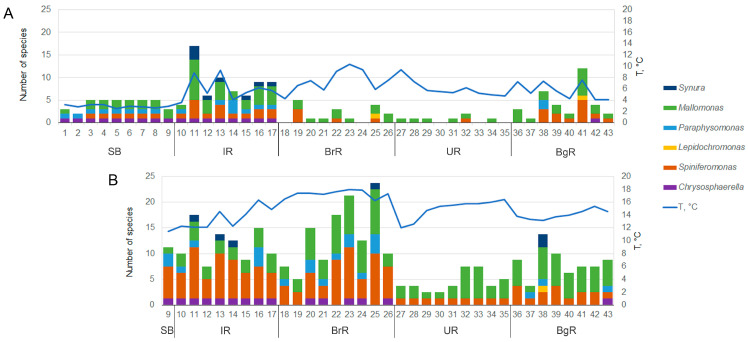
Species richness and structure of silica-scaled chrysophytes by genus, with water temperature. (**A**) Spring 2024. (**B**) Summer 2024.

During the hydrological spring, a total of 30 species were recorded (Figure 12), but richness was unevenly distributed (Figure 13A). Only five species occurred in SB with nearly identical assemblages at stations 3–8 and slightly lower richness was revealed at left-bank sites (stations 1, 2, and 9). The IR supported the richest community (23 species), and all five species present in SB also occurred in IR (Figure 12), reflecting strong species continuity. Moreover, only in SB and IR did four genera—*Chrysosphaerella*, *Spiniferomonas*, *Paraphysomonas*, and *Mallomonas*—co-occur. Species richness increased downstream from SB to IR, accompanied by the appearance of *Synura* (Figure 13A). In the warm bays of IR, richness within *Spiniferomonas* and *Mallomonas* particularly high.

Despite water temperatures favorable for development of silica-scaled chrysophytes, BrR and UR contained only 7 and 2 species, respectively. The BrR assemblage retained with IR: 4 of 7 species were also present in IR (Figure 12). BgR hosted 15 species, forming an assemblage different from the upstream UR, dominated by *Spiniferomonas* and *Mallomonas* (Figure 13A). In spring, scales or cells of *Spiniferomonas bourrellyi*, *S. cuspidata*, *S. trioralis*, *Mallomonas acaroides*, and *M. alpina* were encountered most frequently across the system.

In summer, a total of 33 species were identified. Richness increased in all reservoirs, except IR, which remained the coldest of the downstream systems. Species continuity among reservoirs was more pronounced than in spring (Figure 12). Assemblages in SB (station 9), IR and BrR were especially similar (Figure 13B), all containing *Chrysosphaerella*, *Paraphysomonas*, *Spiniferomonas*, and *Mallomonas* with the latter two genera dominating. BrR yielded the highest number of cells of silica-scaled chrysophytes. UR again exhibited the lowest species richness, with only eight species, seven of which also occurred in BrR. In several BgR stations, the species richness and assemblage closely resembled those of SB, IR, and BrR.

During summer, frequent taxa included *Spiniferomonas cornuta*, *Paraphysomonas gladiata*, *Mallomonas acaroides* forma, *M. akrokomos*, *M. crassisquama*, *M. tonsurata*, *M.* cf. *caudata*. Cells of *Spiniferomonas cuspidata*, *M. acaroides*, and *M. alpina* were also abundant, although these species had predominated in spring. Stomatocyst formation was observed in *Chrysosphaerella coronacircumspina* (Figure 3E), *Spiniferomonas bourrellyi* (Figure 11C,G), *S. cuspidata*/*S. trioralis* (Figure 11E,F), *M. crassisquama* (Figure 11B), and *M. akrokomos* (Figure 11A,D).

In summary, spring assemblages in SB and IR were most similar, with *Mallomonas* and *Spiniferomonas* dominating richness in IR bays and BgR. BrR and UR, despite favorable temperatures, remained relatively species-poor, often yielding only isolated scales. In contrast, summer assemblages across all reservoirs were consistently dominated by *Mallomonas* and *Spiniferomonas*. The closest structural similarity occurred among SB, IR, and BrR, while BgR harbored stations comparable to these upstream systems.

### 3.3. Community Clustering and Ordination

**Figure 14:**
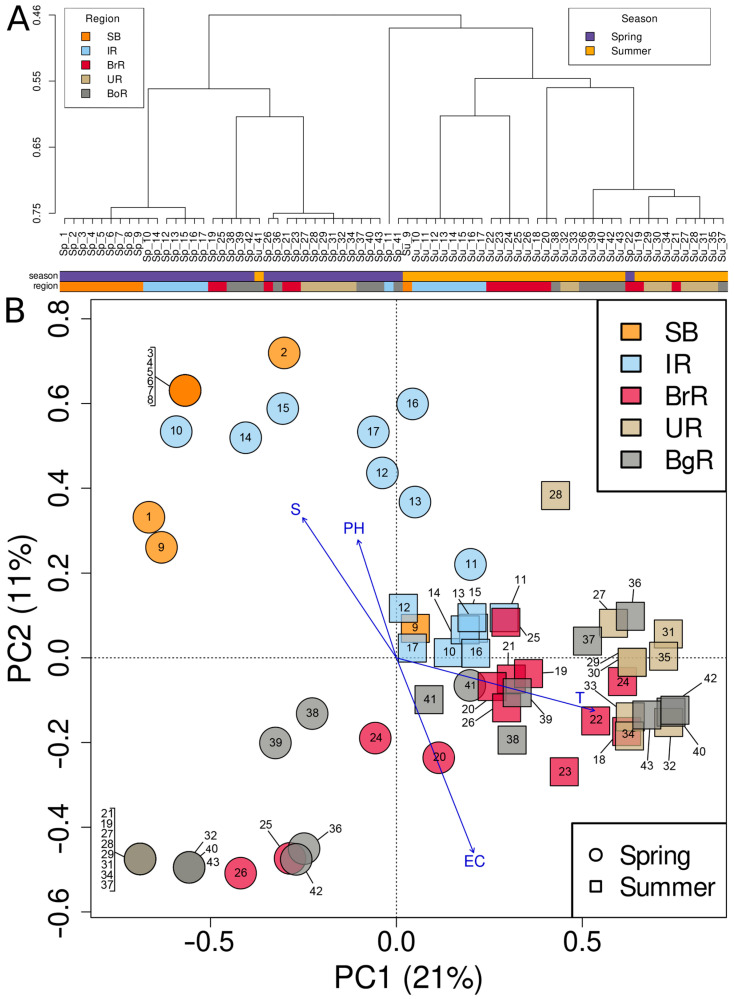
Community similarity patterns based on Bray–Curtis dissimilarities. (**A**) Cluster analysis using affinity propagation; color bars below indicate regional and seasonal affiliation of samples. (**B**) Principal Coordinates Analysis (PCoA) based on presence/absence data. Spring and summer samples are shown as circles and squares, respectively; colors denote regions. Arrows indicate abiotic variables (*p* < 0.001).

Community clustering based on Bray–Curtis dissimilarities with affinity propagation revealed a clear seasonal segregation of assemblages (Figure 14A). Within spring assemblages, clustering clearly separated SB and IR from the northern reservoirs (BrR, UR, and BgR). All stations from the northern reservoirs grouped into a single major branch, but their internal subdivision did not strictly follow reservoir boundaries. Instead, stations from different northern reservoirs were intermixed, indicating that community structure in spring was shaped by basin-wide environmental gradients rather than by individual reservoir identity. Within summer assemblages, IR stations (Su_9–Su_17) still formed a compact group, and a subset of BrR stations (Su_22–Su_26) also clustered together. In contrast, most stations from UR and BgR, together with additional BrR profiles, appeared in mixed subclusters, reflecting greater heterogeneity and weaker alignment with reservoir boundaries compared to spring. Several exceptions crossed the seasonal boundary: Su_41 fell into the spring northern cluster, while Sp_11, Sp_41, and Sp_22 grouped with summer samples.

Principal Coordinates Analysis (PCoA) (Figure 14B) supported these results. The first two axes explained 20.7% and 11.2% of the variance, respectively. Spring samples were positioned on the negative side of PC1, while summer samples occupied the positive side. Spring assemblages showed a broader dispersion along PC2, indicating higher internal heterogeneity, whereas summer assemblages were more compact. The two exceptional cases (Sp_11, Sp_41, and Sp_22) were again placed near the opposite seasonal clusters, consistent with the clustering outcome (Figure 14A).

Environmental fitting further indicated that several abiotic variables were significantly associated with community structure (*p* < 0.001). Among them, temperature (r^2^ = 0.79) and conductivity (r^2^ = 0.67) showed the strongest correlations with the ordination pattern, followed by water transparency (r^2^ = 0.45) and pH (r^2^ = 0.23). These vectors largely aligned with the seasonal gradient along PC1, suggesting that thermal regime and related physicochemical factors were the primary drivers of community separation between spring and summer.

### 3.4. Rare Species and Morphological Features of Individual Species

Rare species *Spiniferomonas crucigera* (Figure 3F) and *Paraphysomonas vacuolata* (Figure 5I) were recorded in the studied reservoir ecosystems, thereby expanding their known biogeography.

*S. crucigera* was originally described from water bodies in Japan [30] and since been reported from reservoirs in Finland [31,32,33,34], Denmark [35], North America [36], Central Russia [37], and Eastern Siberia [38], at pH 4.8–8.4 and T 10–16 °C. In this study, the species was found in BrR at pH 8.1 and T 20.3 °C.

*P. vacuolata* was described from water bodies in Denmark [39] and has been reported from reservoirs in England [40], Finland [39,41], the Netherlands [42], the Baltic Sea [43], and the Southern Urals, Russia [44], at pH 7.6–8.5 and T 14.7–20.5 °C. Here, the species was found in IR at pH 8.5 and T 20.4 °C.

Cells and individual scales of *Mallomonas caudata* and *M. acaroides* with atypical morphology were also observed, which may reflect variation among populations or the presence of cryptic species. Numerous small papillae were found on the scales of *M.* cf. *caudata* using SEM, covering one-half of the basal plate. Large pores along the distal margin were also observed (Figure 9B,D,F). Notably, these features were indistinguishable by TEM. *M.* cf. *caudata* was found in BrR, UR, and BgR.

Cells and individual scales of *M. acaroides* and *M. acaroides* forma were identified. The scales on the *M. acaroides* forma (Figure 6F,G,H,I) differ from those of *M. acaroides* (Figure 6A–E) by their narrower shape, a distinct angular V-shaped rib, and a higher degree of silicification of the ribs and mesh, forming a secondary layer on the shield.

Cells with scales corresponding to *M. crassisquama* (Figure 7A,E) were also recorded, as well as scales with papillae on the dome, anterior submarginal ribs, and the V-rib characteristics of *M. crassisquama* var. *papillosa* (Figure 7B,F). However, since cells bearing both scale types—with and without papillae—were observed simultaneously (Figure 7C), the validity of the *M. crassisquama* var. *papillosa* is questionable. A similar case of a cell with two was previously reported from Lake Labynkyr, Yakutia [45]. In this study, scales with and without papillae are treated as belonging to *M. crassisquama*.

## 4. Discussion

### 4.1. Species Diversity

To detect changes in the structure of freshwater phytoplankton under conditions of increasing anthropogenic pressure and climate change, it is important to establish its current diversity. Over the years, three [46], five [47], and 38 species of silica-scaled chrysophytes have been identified in the IR using electron microscopy [19]. In BgR, 24 species were recorded [48]. No previous studies employing electron microscopy had been conducted in BrR and UR. Our survey revealed a high species richness of silica-scaled chrysophytes across a vast area, including SB and the four large artificial lakes of the Angara Cascade, sampled in two seasons. In total, 45 species of silica-scaled chrysophytes were identified in 2024.

A comparison of species diversity in IR between 2023 [19] and 2024 showed pronounced differences in composition and richness. For instance, in 2023 alone we recorded 11 species absent in 2024: *Lepidochromonas* cf. *stephanolepis*, *L*. cf. *canistrum*, *Paraphysomonas bandaiensis*, *Paraphysomonas* sp. 2, *Mallomonas getseniae*, *M*. *grachevii*, *M*. *trummensis*, *Mallomonas* sp. 1, *Synura echinulata*, *S*. *punctulosa*, and *S*. *spinosa* f. *longispina* (Table S2). Scales, which we previously classified as *Synura* sp. 1 and *Synura* sp. 2 [49] were re-identified in 2024 as *Synura petersenii*. Additionally, *Spiniferomonas minuta* was not detected in SB in 2024, although in 2023 we observed its stomatocysts with diagnostic plate scales [50]. Conversely, eight species were found in IR in 2024 that were not recorded in 2023: *Spiniferomonas abei*, *Paraphysomonas acuminata*, *P*. *vacuolata*, *P. corynephora*, *Mallomonas annulata*, *M. heterospina*, *M*. *insignis*, *Synura spinosa* (Table S2). Stomatocysts attributable to *Paraphysomonas circumvallata* were also detected in 2024 [51].

Differences in species composition likely reflect both the timing of spring and summer sampling and interannual dynamics. In 2023, samples collected on 22–26 June yielded 30 species [19,49], whereas those collected on 24–25 May 2024 contained 23 species. In the summer 22 species were detected in samples from August 17–20 2023 [19], compared to 18 species on August 11–12 2024 (Table S2).

Thus, the actual richness of silica-scaled chrysophytes in BrR, UR, and BgR may be higher when assessed over multiple years. Taking into account species detected in 2023, the cumulative richness in interannual perspective is 12 species in SB and 44 in IR. Altogether, 57 species of silica-scaled chrysophytes were documented in SB and the Angara Cascade reservoirs across 2023 and 2024 (Table S2).

### 4.2. Spatial and Temporal Gradients of Environmental Factors and Dynamics of Silica-Scaled Chrysophytes

Ecologically, water temperature and electrical conductivity provide a mechanistic backdrop for the community patterns observed in the study (Figure 13). The basin-wide summer warming (+11–14 °C) and the rise in EC from spring to summer in upstream regions (SB and IR) imply that the intensity of thermal stratification and mineral supply jointly shape seasonal habitat conditions, especially in the upper and middle reservoirs. The consistent downstream increase in electrical conductivity (and the concomitant decrease in pH), observed in both seasons, accounts for the persistent longitudinal differences in community composition and richness. The light environment further differentiates the lake-to-cascade transition: persistently high water transparency in SB contrasts with lower values in BrR/BgR, consistent with stronger mixing, allochthonous inputs, and summer blooms in reservoirs. Notably, opposite seasonal water transparency trends in IR (spring clearer) and BrR (summer clearer) point to region-specific hydrology and morphology (e.g., residence time, bays) that modulate how temperature and mineralization translate into underwater light regimes. Taken together, these environmental patterns—dominant seasonal shifts in temperature (and upstream in EC), robust longitudinal gradients in EC and pH, and the persistently higher water transparency in SB—provide a coherent physical–chemical framework for the observed seasonal segregation and spatial heterogeneity of assemblages of silica-scaled chrysophytes across the Baikal–Angara Cascade (Figure 14).

**Figure 15:**
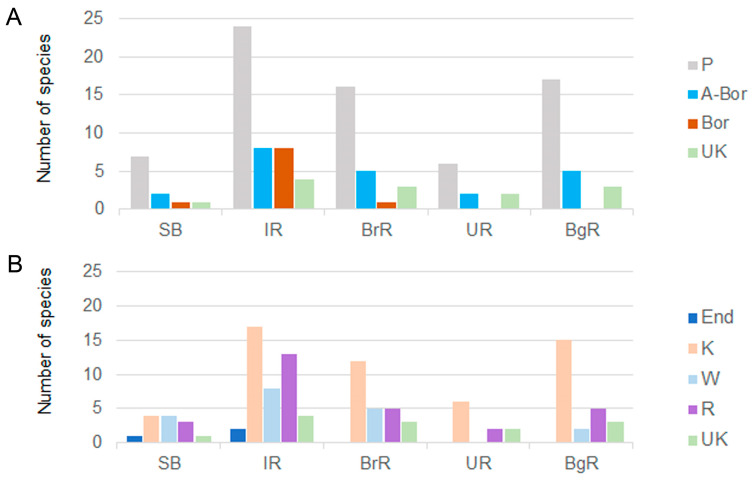
Geographical distribution of silica-scaled chrysophytes. (**A**)—Latitudinal groups. (**B**)—Longitudinal groups.

It is well established that silica-scaled chrysophytes respond sensitively to water temperature changes, which may restructure their assemblage composition and richness. Previous work already revealed temperature-linked shifts in SB and the IR [19,49], and our 2024 data confirm these patterns in the same regions. In contrast, no consistent temperature-richness correlation was detected in downstream northern reservoirs (BrR, UR, BgR). IR showed both higher richness and earlier seasonal shifts compared to northern reservoirs, consistent with its direct exposure to Baikal inflow. This position at the sharpest temperature gradient between SB and IR creates an ecotonal “edge effect”, where species richness and relative abundance are locally enhanced [19]. Analysis of the geographical distribution of silica-scaled chrysophytes further supports this interpretation: IR hosted the highest number of arctic–boreal and boreal species in latitudinal (Figure 15A) and rare in the longitudinal (Figure 15B) groups, with diversity declining downstream.

Notably, two taxa regarded as Baikal endemics—*Chrysosphaerella baikalensis* and *Mallomonas grachevii*—were detected exclusively in IR. In contrast, no boreal species has been recorded in the downstream reservoirs (UR and BgR), which are farthest from Baikal along latitudinal axis. Together, these patterns indicate that IR stand out not only for its highest species richness, but also for the greatest geographic breadth of the silica-scaled chrysophytes assemblage.

Only SB, IR, and BgR exhibited bimodal seasonal dynamics in the species richness of silica-scaled chrysophytes, with spring and summer peaks. Across these reservoirs, taxa fell into three phenological groups: spring-restricted, summer-restricted, and persistent (occurring in both seasons) (Table S2). In BrR, no spring increase was detected despite favorable temperatures; instead, a single pronounced summer maximum occurred (Figure 13), accompanied by shifts in species composition from spring to summer (Figure 12). Five of the six spring taxa reappeared in summer in BrR, consistent with turnover rather than wholesale replacement. UR showed consistently low richness in both seasons despite suitable hydrochemistry. Its assemblage comprised scattered occurrences of one cosmopolitan and one rare species of the genera *Mallomonas* and *Spiniferomonas*, suggesting hydrological constraints (e.g., higher main-channel velocities) that suppress other, more demanding genera. This interpretation accords with prior observations that the strong currents limit *Chrysosphaerella*, *Paraphysomonas*, *Lepidochromonas*, *Spiniferomonas*, and *Synura* [52], whereas tributaries and large bays showed the increased richness of silica-scaled chrysophytes along large rivers [52,53].

### 4.3. Features of the Formation of the Species Composition in the Reservoirs of the Angara Cascade

The diversity of silica-scaled chrysophytes in large rivers of the world is practically not studied. The estuaries and deltas of rivers remain the most studied [53,54,55,56,57]. Rheophilic conditions generally constrain flagellated microeukaryotes, particularly fragile scaled forms, because high current velocities inhibit their persistence. However, in impoundment systems the rugged reservoir shoreline generates shallow, well-warmed bays with reduced or absent flow, creating habitats favorable for development of silica-scaled chrysophytes [52]. Hydropower dams further accentuate these conditions by slowing river discharge, thereby facilitating colonization and diversification of scaled chrysophytes.

Biogeographical analysis (Figure 15) revealed that diversity in the Angara Cascade reservoirs is shaped predominantly by latitudinal polyzonal (64%) and longitudinal cosmopolitan (42%) species, supplemented by latitudinal arctic–boreal (15%) and widespread (22%) or rare (22%) species, reflecting the regional specifics of the northern reservoirs (Table S2). This structure mirrors patterns reported from other large rivers: in the temperate Ob River, Russia, among 67 species of silica-scaled chrysophytes polyzonal and cosmopolitan species, such as *Mallomonas acaroides*, *M. alpina*, *M. akrokomos*, *M. crassisquama*, and *M. tonsurata* are reported [52]. Similarly, in the subtropical Paraná Delta, Argentina, 33 polyzonal and cosmopolitan taxa, such as *Mallomonas alpina, M. akrokomos*, and *M. tonsurata,* are present but occur at lower abundance [57]. In contrast to subtropical rivers, the Angara reservoirs lack tropical elements but support several arctic–boreal taxa, underscoring the climatic specificity of northern regulated rivers and their sensitivity to environmental change.

Beyond geographical range, trophic differentiation also contributes to the structuring of assemblages of silica-scaled chrysophytes. Of the 57 species recorded, 44% are phototrophs and 31% mixotrophs, both groups contributing to the Si cycle and primary production, while ~25% are heterotrophs that do not produce organic matter. Auto- and mixotrophs (mainly *Spiniferomonas* and *Mallomonas*) were ubiquitous across reservoirs in both seasons, but heterotrophs (*Paraphysomonas* and *Lepidochromonas*) displayed distinct seasonal and spatial patterns. Most heterotrophs are boreal taxa described from temperate waters [16,58,59], and in our study their diversity peaked in summer when the water temperatures were highest. Eight heterotrophic species were summer-restricted, three occurred only in spring (*L. takahashii*, *P. corynephora* and *Paraphysomonas* sp. 2), and two were present in both seasons (*P. uniformis* subsp. *hemiradia* and *Paraphysomonas* sp. 3). Over two years, IR—characterized by the steepest thermal gradient—harbored the greatest heterotrophic diversity (10 species) (Table S2), suggesting that its transitional position between Baikal and downstream reservoirs enhances niche heterogeneity.

Overall, the species composition of the Angara Cascade reservoirs reflects the combined effects of flow regulation, climatic setting, and trophic diversity. Polyzonal and cosmopolitan elements provide the backbone of communities, while the presence of arctic–boreal and heterotrophic taxa highlights both the regional uniqueness and the ecological complexity of these northern impoundments.

## 5. Conclusions

This study represents the first detailed SEM/TEM-based survey of silica-scaled chrysophytes in the Angara Cascade reservoirs and Southern Baikal. We documented 57 species across two years, with strong seasonal contrasts and Irkutsk Reservoir emerging as both a hotspot of richness and a transitional ecotone. The assemblages combine cosmopolitan and polyzonal taxa with Baikal endemics and arctic–boreal species, reflecting both global patterns and regional climatic specificity.

The coexistence of phototrophic, mixotrophic, and heterotrophic chrysophytes highlights their functional importance in silica cycling and primary production. Reservoir regulation created diverse habitats that sustain high richness, while the persistence of endemic and boreal taxa indicates sensitivity to climate and hydrological shifts. These findings underscore the applied value of SEM/TEM-based monitoring and establish a baseline for biodiversity assessment in large, regulated river systems.

## Data Availability

The data presented in this study are original and were collected specifically for this research. They are not openly available in a public repository but are available from the corresponding author upon reasonable request and with the necessary considerations for participant confidentiality and ethical restrictions.

## Supplementary Materials

The following supporting information can be downloaded at https://www.mdpi.com/article/10.3390/biology14101325/s1. Table S1. Sampling sites in Southern Baikal and Angara Cascade reservoirs and their environmental parameters in 2024 (site numbers according to a Figure 1). Table S2. The species composition of silica-scaled chrysophytes in Southern Baikal (SB) and four reservoirs located downstream of the Angara River: Irkutsk (IR), Bratsk (BrR), Ust-Ilimsk (UR), and Boguchany (BgR) in 2024. Spring—species was discovered only in spring; Summer—species was discovered only in summer; Spring + Summer—species is found in spring and summer. (*)—noted species that expanded their spring diversity in Southern Baikal and the Irkutsk Reservoir in 2024. (**)—noted species found in Southern Baikal and the Irkutsk Reservoir only in 2023 [19], and not observed in 2024.
