# Supplementary material for: High Diversity and Spatiotemporal Dynamics of Silica-Scaled Chrysophytes (Class Chrysophyceae) in Reservoirs of the Angara Cascade of Hydroelectric Dams
Source: Biology (Basel). 2025 Sep 25;14(10):1325. doi: 10.3390/biology14101325 (PMC12561758; doi:10.3390/biology14101325)
Supplement: Supplementary file 1 [file biology-14-01325-s001.zip › Supplement_Table S1.pdf]

Table S1. Sampling sites in Southern Baikal and Angara Cascade reservoirs and their environmental parameters in 2024 (site numbers according to a Figure 1).

Station numbers	Reservoirs	Coordinates N/E	Date of sampling dd.mm.yyyy	S, m	pH	T, °C	EC, mS/m	Date of sampling dd.mm.yyyy	S, m	pH	T, °C	EC, mS/m
1	Southern Baikal, SB	51°40.578/103°52.309	24.05.2024	12	8.03	3.23	97	-	-	-	-	-
2		51°45.546/104°13.222	24.05.2024	9.5	8.33	2.85	95	-	-	-	-	-
3		51°38.710/104°13.715	24.05.2024	10.5	8.17	3.2	96	-	-	-	-	-
4		51°31.428/104°14.417	24.05.2024	10.5	8.21	3.26	94	-	-	-	-	-
5		51°36.402/104°44.147	24.05.2024	11.5	8.33	2.58	94	-	-	-	-	-
6		51°35.440/105°06.968	25.05.2024	11	8.3	2.95	89	-	-	-	-	-
7		51°46.731/105°22.528	25.05.2024	10.5	8.27	2.82	88	-	-	-	-	-
8		51°42.262/105°00.720	25.05.2024	11.5	8.27	2.66	108	-	-	-	-	-
9		51°49.033/104°54.616	25.05.2024	13	8.31	2.91	98	11.08.2024	10	8.41	14.36	112
10	Irkutsk Reservoir, IR	52°02.522/104°35.565	26.05.2024	9	8.35	3.65	92	11.08.2024	7	8.45	15.34	111
11		52°03.839/104°27.439	26.05.2024	4	8.5	8.85	100	11.08.2024	-	8.6	15.1	112
12		52°06.984/104°28.510	26.05.2024	5	8.16	5.2	102	11.08.2024	5	8.55	15.1	112
13		52°10.189/104°29.452	26.05.2024	4	8.36	9.3	105	12.08.2024	4.5	8.59	18.2	112
14		52°08.388/104°27.439	26.05.2024	9.5	8.37	4.1	104	12.08.2024	5.5	8.47	15.37	107
15		52°13.204/104°21.934	27.05.2024	10.5	8.33	5.33	91	12.08.2024	5	8.5	17.7	113
16		52°11.869/104°21.146	27.05.2024	4	8.52	6.2	91	12.08.2024	4	8.54	20.45	117
17		52°14.486/104°19.802	27.05.2024	7.5	8.4	5.7	91	12.08.2024	3.5	8.54	18.64	114

18	Bratsk Reservoir, BrR	56°10.981/102°18.693	04.06.2024	7	7.64	4.36	146	24.08.2024	8.5	8.12	20.6	164
19		56°07.309/102°05.097	04.06.2024	4	7.97	6.6	145	24.08.2024	10	8.2	21.7	166
20		55°57.321/101°52.738	03.06.2024	4.5	8.29	7.5	131	23.08.2024	6.5	8.29	21.7	166
21		55°52.703/101°40.708	04.06.2024	4	7.87	5.8	143	24.08.2024	6.5	8.27	21.5	166
22		55°38.051/102°01.636	03.06.2024	4	8.48	9.1	137	24.08.2024	4	8.1	22	154
23		55°31.671/102°21.412	03.06.2024	2.5	8.47	10.4	140	23.08.2024	3.5	8.09	22.4	144
24		55°31.717/102°08.723	03.06.2024	2	8.24	9.4	145	23.08.2024	4	8.1	22.3	151
25		56°04.736/101°48.535	03.06.2024	4	7.96	5.9	140	24.08.2024	6.5	8.1	20.3	167
26		56°15.184/101°45.734	04.06.2024	6.5	8.14	7.6	145	24.08.2024	8	8.27	21.6	168
27	Ust-Ilim Reservoir, UR	57°09.135/102°20.423	07.06.2024	5.5	8.01	9.4	154	29.08.2024	4.5	7.23	15	152
28		57°20.124/102°20.588	07.06.2024	5.5	7.95	7.26	154	29.08.2024	5	6.97	15.7	151
29		57°28.336/102°23.225	07.06.2024	6	7.68	5.7	155	29.08.2024	5.5	7.46	18.43	151
30		57°34.221/102°23.719	06.06.2024	7	7.64	5.5	154	29.08.2024	5.5	7.39	19.2	150
31		57°39.209/102°30.888	06.06.2024	8	7.57	5.23	155	29.08.2024	5.5	7.68	19.4	154
32		57°40.002/102°36.326	06.06.2024	6.5	7.56	6.2	158	28.08.2024	5.5	7.73	19.7	148
33		57°46.075/102°37.644	06.06.2024	7	7.49	5.23	155	28.08.2024	5.5	7.54	19.6	149
34		57°52.197/102°40.528	06.06.2024	6	7.59	4.9	151	28.08.2024	6	8.29	19.9	148
35		57°57.160/102°40.853	06.06.2024	6	7.31	4.8	158	28.08.2024	7	8.28	20.5	150
36	Boguchany Reservoir, BgR	58°37.572/100°33.883	11.06.2024	4.5	8.08	7.3	184	01.09.2024	4	7.24	17.36	187
37		58°18.454/100°18.217	11.06.2024	6	7.64	5.2	181	01.09.2024	4	7.08	16.73	188
38		58°21.391/99°59.647	11.06.2024	5.5	7.4	7.4	180	01.09.2024	4.5	7.08	16.53	175
39		58°32.805/99°31.426	11.06.2024	5.5	7.29	5.6	179	01.09.2024	4	7.2	17.23	176

40		58°31.945/99°45.763	09.06.2024	4.5	7.34	4.3	174	31.08.2024	4.5	7.16	17.5	175
41		58°36.500/99°23.516	09.06.2024	3	7.56	7.6	180	31.08.2024	3	7.21	18.23	174
42		58°36.521/99°23.475	09.06.2024	6	7.29	4.1	174	31.08.2024	4	7.34	19.23	174
43		58°41.965/99°13.546	09.06.2024	5	7.24	4.1	177	31.08.2024	3	7.28	18.26	172
