# Supplementary material for: High Diversity and Spatiotemporal Dynamics of Silica-Scaled Chrysophytes (Class Chrysophyceae) in Reservoirs of the Angara Cascade of Hydroelectric Dams
Source: Biology (Basel). 2025 Sep 25;14(10):1325. doi: 10.3390/biology14101325 (PMC12561758; doi:10.3390/biology14101325)
Supplement: Supplementary file 1 [file biology-14-01325-s001.zip › Supplement_Table S2.pdf]

Table S2. The species composition of silica-scaled chrysophytes in Southern Baikal (SB) and four reservoirs located downstream of the Angara River: Irkutsk (IR), Bratsk (BrR), Ust-Ilim (UR), and Boguchany (BgR) in 2024. Spring – species was discovered only in spring; Summer – species was discovered only in summer; Spring+Summer – species is found in spring and summer. (*) – noted species that expanded their spring diversity in Southern Baikal and the Irkutsk Reservoir in 2024. (**) – noted species found in Southern Baikal and the Irkutsk Reservoir only in 2023 [19], and not observed in 2024.

No.	Geographical distribution		Trophic mode	Taxon	Reservoirs				
	Longitudinal	Latitudinal			SB	IR	BrR	UR	BgR
**1.**	Bor	End	Mix	*Chrysosphaerella baikalensis* Popovskaya	Spring	Spring			
2.	P	K	Mix	*C. coronacircumspina* Wujek et Kristiansen	Summer	Summer	Summer		
3.	P	K	Mix	*C. brevispina* Korshikov		Spring	Summer		Spring+ Summer
4.	P	W	Mix	*Spiniferomonas abei** Takahashi		Summer			
5.	A-Bor	R	Mix	*S. abrupta* Nielsen		Summer			Spring
6.	P	W	Mix	*S. bilacunosa* Takahashi			Summer		
7.	P	K	Mix	*S. bourrellyi* Takahashi		Summer	Spring+ Summer	Spring	Spring+ Summer
8.	A-Bor	R	Mix	*S. cornuta* Balonov	Summer	Summer	Summer	Summer	Spring+ Summer
9.	P	R	Mix	*S. conica* Takahashi			Summer		

10.	A-Bor	R	Mix	*S. crucigera* Takahashi			Summer		
11.	A-Bor	R	Mix	*S. cuspidata* (Balonov) Kapustin	Spring+ Summer	Spring+ Summer	Spring+ Summer	Summer	Spring+ Summer
12.	A-Bor	R	Mix	*S. minuta*** Nicholls	Summer				
13.	A-Bor	R	Mix	*S. silverensis* Nicholls		Spring+ Summer	Summer		Summer
14.	P	W	Mix	*S. serrata* Nicholls			Summer		Spring
15.	P	W	Mix	*S. septispina* Nicholls	Summer	Summer	Summer		
16.	A-Bor	R	Mix	*S. triangularis* Siver		Spring			
17.	P	K	Mix	*S. trioralis* Takahashi	Summer	Spring+ Summer	Spring	Summer	Spring+ Summer
18.	Bor	R	Mix	*S. takahashii* Nicholls		Summer			
19.	Bor	R	Hetero	*Lepidochromonas* cf. *canistrum*** (Preisig et Hibberd) Kapustin et Guiry		Summer			
20.	P	K	Hetero	*L. butcheri* (Pennick et Clarke) Kapustin et Guiry					Summer
21.	Bor	R	Hetero	*L.* cf. *stephanolepis*** (Preisig et Hibberd) Kapustin et Guiry		Summer			
22.	P	K	Hetero	*L. takahashii* (Cronberg et Kristiansen) Kapustin et Guiry			Spring		Spring
23.	P	W	Hetero	*Paraphysomonas acuminata** Scoble et Cavalier-Smith		Summer			Summer
24.	Bor	R	Hetero	*P. bandaiensis*** Takahashi		Summer			
25.	P	W	Hetero	*P. circumvallata** Wujek	Summer				

26.	P	W	Hetero	*P. corynephora** Preisig et Hibberd	Spring	Spring			
27.	P	K	Hetero	*P. gladiata* Preisig et Hibberd		Summer	Summer		Summer
28.	P	K	Hetero	*P. uniformis* subsp. *hemiradia* Scoble et Cavalier-Smith		Spring	Summer		Spring
29.	Bor	R	Hetero	*P. vacuolata** Thomsen		Summer			
30.	UK	UK	Hetero	*Paraphysomonas* sp. 1	Summer				
31.	UK	UK	Hetero	*Paraphysomonas* sp. 2**		Spring			
32.	UK	UK	Hetero	*Paraphysomonas* sp. 3		Spring	Summer		Summer
33.	P	K	Photo	*Mallomonas acaroides* Perty		Spring+ Summer	Spring+ Summer	Summer	Spring+ Summer
34.	UK	UK	Photo	*M. acaroides* forma			Summer	Summer	Summer
35.	P	K	Photo	*M. akrokomos* Ruttner			Spring+ Summer	Summer	Spring+ Summer
36.	P	K	Photo	*M. alpina* Pascher et Ruttner	Spring+ Summer	Spring+ Summer	Spring+ Summer	Spring	Spring+ Summer
37.	P	K	Photo	*M. annulata** Harris		Spring			Spring
38.	UK	UK	Photo	*M.* cf. *caudata* (Ivanov) Krieger			Summer	Summer	Summer
39.	P	K	Photo	*M. crassisquama* (Asmund) Fott		Spring+ Summer	Summer	Summer	Summer
40.	A-Bor	R	Photo	*M. getseniae*** (Voloshko) Bessudova		Spring			
41.	Bor	End	Photo	*M. grachevii*** Bessudova		Spring			

42.	P	K	Photo	*M. heterospina** Lund		Spring			Spring
43.	P	W	Photo	*M. insignis** Penard		Spring			
44.	A-Bor	R	Photo	*M. multiunca* Asmund					Spring
45.	P	K	Photo	*M. punctifera* Korshikov		Spring			
46.	P	K	Photo	*M. striata* Asmund		Spring			
47.	P	K	Photo	*M. tonsurata* (Teiling) Krieger		Spring+ Summer	Summer		Summer
48.	A-Bor	R	Photo	*M. trummensis*** Cronberg		Spring			
49.	P	W	Photo	*M. vannigera* Asmund	Spring	Spring	Summer		
50.	UK	UK	Photo	*Mallomonas* sp. 1**		Spring			
51.	UK	UK	Photo	*Mallomonas* sp. 2		Spring			
52.	P	K	Photo	*Synura echinulata*** Korshikov		Spring			
53.	Bor	W	Photo	*S. glabra* (Korshikov) Škaloud et Kynclová		Spring+ Summer	Summer		
54.	P	K	Photo	*S. petersenii* (Korshikov) Škaloud et Kynčlová		Spring			Summer
55.	A-Bor	R	Photo	*S. punctulosa*** Balonov		Spring			
56.	P	K	Photo	*S. spinosa** Korshikov		Spring			
57.	P	W	Photo	*S. spinosa* f. *longispina*** Petersen et Hansen		Spring			
				Total	12	44	25	10	25
